# Hypoxia Induced Factor in Chronic Kidney Disease: Friend or Foe?

**DOI:** 10.3389/fmed.2017.00259

**Published:** 2018-01-22

**Authors:** Weiying Li, Yuliang Zhao, Ping Fu

**Affiliations:** ^1^Division of Nephrology, West China Hospital, Sichuan University, Chengdu, China

**Keywords:** erythropoiesis-stimulating agents, chronic kidney disease-related anemia, chronic kidney disease, hypoxia-induced factor, erythropoietin, prolyl hydroxylase domain inhibitors, prolyl hydroxylase domain, roxadustat

## Abstract

Many studies have shown evidence that erythropoiesis-stimulating agents (ESAs), as a classic treatment for chronic kidney disease (CKD)-related anemia, have several disadvantages and may trigger various adverse events with long-term use. The hypoxia-induced factor (HIF) pathway has been intensively investigated in kidney disease, especially in CKD, as research has shown that HIF-mediated erythropoiesis might work as a potential therapeutic strategy for managing CKD-related anemia. Development of prolyl hydroxylase domain inhibitors (PHIs), as an effective HIF activator, is a valuable step toward finding a replacement for ESAs, which showed an effective erythropoiesis through a comprehensive and physiological approach by promoting erythropoietin production, increasing iron bioavailability and improving chronic inflammatory status. Heretofore no adverse events or obvious off-target effects have been reported in clinical trials of PHIs. Nevertheless, a cautious inspection with extended follow-up period is warranted to validate the safety of prolonged HIF elevation, especially considering its ambiguous role in fibrogenesis and inflammation responses and possible risks in accelerating vascular calcification and tumorigenesis. A weighed dosing strategy might be the key to circumvent the unexpected side-effect brought by pleotropic effects of HIF elevation and achieve a selective augmentation of HIF-mediated signaling pathway. New studies with longer follow-up period and adequate analysis about the risks for proinflammation, vascular calcification and tumorigenesis are needed to ensure the drugs are safe for long-term use before being widely accepted in daily clinical practice.

## Introduction

Hypoxia-induced factor (HIF) pathway has been intensively investigated in kidney disease, especially in chronic kidney disease (CKD), as research has shown that HIF-mediated erythropoietin (EPO) synthesis might work as a potential therapeutic strategy for managing CKD-related anemia. Traditional management for CKD-related anemia includes erythropoiesis-stimulating agents (ESAs) with adjuvant iron supplement. Blood transfusion is considered in ESAs hyporesponsive cases. However, ESAs therapy has many caveats and leads to an increased risk of thrombosis and cardiovascular events (1). Growing evidence shows ESAs were related to overshoot of endogenous EPO and oscillations in hemoglobin levels, therefore increasing risk of myocardial infarction, venous thromboembolism, or mortality risks (2). At the same time, the in-depth investigation shed light on the mechanism of CKD-related anemia, giving more insight about anemia in ESRD patients being more than an “EPO deficient” status, but being an outcome of intricate interactions between the impaired production of EPO, chronic inflammatory status and disrupted iron balance (3). Considering the possible cardiovascular risks brought by ESAs, and a limited benefit in improving chronic inflammatory status and iron dysregulation, an alternative erythropoietic agent with more comprehensive effects and fewer risks is anticipated (1).

Hypoxia-induced factor regulated by prolyl hydroxylase domain-containing proteins (PHDs) plays a key role in EPO expression, and it also has been implicated in regulation of iron bioavailability. HIF-α normally is hydroxylated by PHDs, then ubiquitinated by von Hippel-Lindau tumor suppressor protein (pVHL) and finally degraded by proteasome. Under hypoxia condition, PHDs are inhibited which allows HIF-α to escape the hydroxylation by PHDs and to be stabilized. Accumulated HIF-α forms a heterodimer with β subunits, which is constitutively expressed, and enters the nucleus as a transcriptional factor. It binds to hypoxia response element and regulates the expression of multiple target genes, participating in a wide range of pathophysiological processes, including renal fibrosis, anemia, inflammation, vascular calcification and angiogenesis (4). Recently, applying prolyl hydroxylase domain inhibitors (PHIs) to elevate HIF and to treat CKD-related anemia has become a highly appealing research area for nephrologists and scholars. HIF facilitates intestinal iron absorption by promoting the expression of divalent metal transporter 1 and duodenal cytochrome B (5), stimulates erythropoiesis by upregulating endogenous EPO production, enhances iron uptakes by proerythrocytes by raising the expression transferrin receptor, and propels the recycling of iron in phagocytosed erythrocytes by modulating heme-oxygenase-1. HIF could also improve chronic inflammatory status by elevating erythroferrone and inhibiting hepcidin (6). Compared with ESAs, PHIs, as HIF stabilizers, demonstrated good effectiveness in treating CKD-related anemia with a comprehensive effect on replenishing endogenous EPO, decreasing hepcidin, and improving the bioavailability of iron. Appealingly, PHIs might reduce the cardiovascular risks by maintaining a physiological elevation of EPO and avoiding hemoglobin overshoot brought by ESAs. PHIs-mediated augmentation of HIF pathway appears promising and has triggered robust research on the clinical prospect. Several PHIs have been under development, such as Roxadustat (FG-4592), Vadadustat, Molidustat, GSK1278863, JTZ-951, and DS-1093a (7). In a randomized, placebo-controlled phase 2a clinical trial, Roxadustat transiently and moderately increased endogenous EPO and reduced hepcidin level in nondialysis-dependent (NDD) CKD patients with an adverse events rate similar to placebo during the (up to) 12-week follow-up period (8). The efficacy and tolerability of Roxadustat were also verified in anemic NDD CKD patients (9), incident dialysis patient (10, 11), and in dialysis-dependent patients who were on epoetin alfa before (12), however, all the clinical trials published had a short follow-up period. The EPO-promoting effect of PHIs was independent from baseline CRP and iron deficiency status. Similar promising results were reported in clinical trials about Vadadustat and GSK1278863 (13, 14). All these evidences from clinical trials appear inspiring, bringing hope that PHIs might be a better option than ESAs in raising hemoglobin and treating CKD-related anemia.

However, preclinical studies illustrated that the role of HIF in pathogenesis of kidney disease is highly context dependent (15). Whether HIF has a profibrotic or antifibrotic effect is still disputable. Hill used genetic approach to downregulate HIF expression, which led to more severe histologic damage after the exposure to acute ischemic insult. In comparison, chemical activation of HIF before undergoing acute renal ischemia by L-mimosine showed a better persevered tubular morphology and protected mouse kidneys from ischemic injury (16). Bernhardt et al. applied a PHI FG-4487 before imposing ischemic injury and subsequently observed attenuated tubular damage and improved kidney function (17). Pinelopi et al. found preischemic targeting of HIF by PHIs ameliorated fibrosis by protecting against cell death in acute ischemic injury, however, postischemic application of PHIs failed to show similar renoprotective effects (18). Administration of HIF prior to insult propels acclimation to hypoxia by triggering a series of metabolic adjustments toward stress. Xiaofang et al. also demonstrated a short-term HIF activation by L-mimosine 4 weeks after subtotal nephrectomy reduced the expression of fibrosis markers and improved renal function (19). It indicated that an appropriate and short-term augmentation of HIF—even imposed after injury—might boost a previously suboptimal elevation of HIF and promote cellular adaption to hypoxia, though some studies reported that postconditional HIF induction failed to show renoprotective benefits (18). Although a growing body of evidence shows that preconditioned increase of HIF might have a protective role in preserving kidney function and prevent the transition from AKI to CKD, overactivation and prolonged exposure to HIF is implicated as a pathogenic factor in CKD. A basic pathological principle is that hypoxia, as one important cause of cellular injury when its severity exceeds the cell’s ability to adapt, might lead to cell death and maladaptation such as fibrotic repair and scar formation. When applying this principle to renal pathology, we would draw a conclusion consistent with the well-known fact that chronic hypoxia leads to tubulointerstitial fibrosis. Significant decline of renal function with upregulated expression of HIF-1α and HIF-2α resulting from PHD2 deletion was observed in endothelial-specific PHD2 knockout mouse model, which might be mediated by Notch3/growth factor-β1 (TGF-β1) signaling (20). Kimura et al. found that stable expression of HIF-1α by VHL deletion significantly increased renal fibrosis in a 5/6 renal ablation model, and this effect could be rescued by applying anti-HIF-1α agent YC-1 in a unilateral ureteral obstruction (UUO) mouse model (21). Higgins et al. demonstrated that genetic ablation of epithelial HIF-1α inhibited the development of tubulointerstitial fibrosis in UUO kidneys with attenuated interstitial collagen deposition, and that increased HIF-1α expression is associated with tubulointerstitial injury in patients with CKD (22). Mechanistically, HIF promotes extracellular matrix remodeling, fibroblast activation, collagen deposition, and epithelial–mesenchymal transition by interacting with various fibrogenic factors such as collagen type I alpha 2 chain (COL1A2), SMAD3, plasminogen activator inhibitor-1, and tissue inhibitor of metalloproteinase 1, and by crosstalk with many profibrotic signaling pathways, such as TGF, Notch, NF-κB, and PI3K/Akt pathways (23). HIF is a critical contributor to progression of renal fibrosis, which is in sharp contrast with its renoprotective effects when preconditioning in acute injury. They do not necessarily contradict each other if we look deep into the matter. HIF preconditioning with mild hypoxia stress increases the body’s tolerability and resistance to a “real” hypoxia injury. When HIF is activated at a proper level and within a well-regulated extent by pharmacological approach, HIF-prompted cellular adaption could be further optimized and reinforced. It is noted that excessive stress needs to be removed in time with restoration of local oxygen supply before a pathological maladaptation process is initiated. HIF stabilizers create a chronic pseudohypoxia environment by mimicking response to the stress of hypoxia, which would expose cells to elevated HIF and activate downstream HIF-mediated signaling pathways. Theoretically, the extent and duration of pseudohypoxia would be pivotal to the overall effects of HIF activation, and selective activation of HIF-mediated signaling pathways might be PHIs dose dependent. It emphasizes the significance of tightly controlling the extent of HIF activation by weighing the dose of PHIs cautiously to reduce unwanted risks (15). Furthermore, many other factors could also bring discrepancies to the effect of modulating HIF expression. With ubiquitous HIF expression in endothelial, epithelial, interstitial and myeloid cells, changing HIF expression might lead to distinct consequences because it has disparate functions in different cell types, which might also raise the concern about triggering off-target side-effects with universally elevated HIF expression. Variety in methods of modifying HIF might also be a contributing factor. A tissue-specific, complete deletion of PHD or VHL by genetic knock-out approaches might demonstrate its unique effects on HIF-involved pathway network compared with a global and partial modification of HIF expression by pharmacological method (24).

In addition to a disputable role of HIF on the development of renal fibrosis, its association with inflammation, vascular calcification and tumorigenesis is also obscure. Accumulating studies have reported HIF promotes inflammation in a broad range of diseases. A bidirectional interaction was found between inflammation and augmentation of HIF-mediated signaling pathways. Yamaguchi et al. showed that CCAAT/enhancer-binding protein δ, when triggered by inflammatory cytokines, enhanced HIF-1α expression in tubulointerstitial hypoxia and therefore propelled inflammatory response (25). Conversely, change in HIF expression also modulates inflammatory response primarily *via* NF-κB-dependent manner. Genetic deletion of epithelial HIF alleviated infiltration of inflammation cells (22), but a conflicting finding was found when upregulating HIF expression in myeloid cells. Kobayashi et al. found that global or selective activation of HIF in myeloid cells by Cre-loxP recombination suppressed inflammation in mice subjected to UUO (26). Overall, the proinflammatory or anti-inflammatory effect of HIF seems to be tissue-specific and HIF-subtype dependent, and a detailed interaction of HIF-mediated inflammatory pathways in different pathophysiologic scenarios warrants further investigation. Another non-ignorable outcome of prolonged HIF activation is vascular calcification, a chief cause of cardiovascular risks in CKD patients. Although a growing body of evidence showed that HIF preconditioning in myocardial ischemia reperfusion injury might have cardio-protection effects by targeting endothelial nitric oxide synthase and activating prosurvival signaling cascades of Akt, there is a lack of data about the effect of a long-term HIF elevation on heart function and myocardial reconstruction (27). On the other hand, Mokas et al. identified HIF as a procalcifying factor which synergizes with elevated inorganic phosphate to enhance osteogenic transdifferentiation of vascular smooth muscle cell and calcification. HIF activators Roxadustat could further promote vascular calcification, which is the essential cause for CKD-related cardiovascular complications (28). Therefore, whether a potential cardioprotection effect of transient HIF elevation might outweigh its role in promoting vascular calcification with prolonged use remains elusive, and a net effect from these two opposed forces on cardiovascular risks in CKD patients needs further investigation. Using PHIs to elevate HIF level might also arouse a concern as HIF is also a well-known regulator in angiogenesis and tumor development (29). HIF orchestrates the process of angiogenesis and neovascularization by transcriptionally targeting various angiogenic factors such as VEGF, angiopoietin 2 and a Notch ligand, delta-like ligand 4 and by regulating proangiogenic chemokines. However, dysregulated angiogenesis with excessive activation of VEGF and improper neovascularization could be strong driving force for tumor progression and metastasis (30). In theory, potential risks of tumorigenesis with long-term application of HIF activators could be a concern unless being proven otherwise.

In summary, several clinical trials reported that PHIs, as HIF stabilizers had a good performance in increasing the production of EPO, decreasing the level of hepcidin and improving chronic inflammatory status. It also demonstrated potential benefits on improving total cholesterol profile and blood pressure-lowering effect in ESRD patients. Clinical trials reported decreased cardiovascular risks of PHIs in CKD patients, possibly mediated by avoiding EPO overshoots and oscillation of hemoglobin. Therefore, PHIs might be a promising alternative for ESAs in treating CKD-related anemia. However, the effects of HIF elevation are pleiotropic and context dependent, which contribute to the disputed role of HIF manipulation in CKD. Transient HIF elevation during the early stage of injury contributes to hypoxia acclimation. Nevertheless, long-term immoderate hypoxia with prolonged HIF elevation might turn out to be deleterious. An inappropriate activation of HIF is associated with exacerbation of fibrogenesis and deterioration of kidney function, predisposing kidney to a pathological microenvironment that mimics long-term hypoxia. Disparity was also observed in its effects on inflammation, which added to the complexity of HIF activation therapy. These discrepancies might be partially explained by the difference in time and duration of intervention administration, variety in lab methods of modulating HIF expression and its tissue-specific property. The perceived benefits on cardio-protection of HIF augmentation must be balanced against a potential risk of vascular calcification, which warrants further investigation. These ambiguities and theoretical risks of tumorigenesis call for studies with longer follow-up period and cautious titer of PHIs doses to achieve a satisfying selective activation of HIF-mediated signaling pathway and to minimize unexpected adverse effects. Careful examination of the effect of PHIs on the kidney function of less advanced CKD patients or NDD CKD patients in clinical trials with long term use is especially important, considering its potential profibrotic effects might exacerbate the already-compromised kidney function. The significance of applying PHIs transiently before acute kidney injury or kidney transplant to achieve HIF preconditioning and hypoxia acclimation is worth further evaluation.

## Author Contributions

WL took the major in conceptualization, literature search and review, article drafting, and writing. PF contributed by general supervision, reviewing and validation. YZ contributed by editing and reviewing.

## Conflict of Interest Statement

The authors declare that the research was conducted in the absence of any commercial or financial relationships that could be construed as a potential conflict of interest.
